# Metabolomics integrated with network pharmacology of blood-entry constituents reveals the bioactive component of Xuefu Zhuyu decoction and its angiogenic effects in treating traumatic brain injury

**DOI:** 10.1186/s13020-024-01001-0

**Published:** 2024-09-26

**Authors:** Teng Li, Lianglin Zhang, Menghan Cheng, En Hu, Qiuju Yan, Yao Wu, Weikang Luo, Hong Su, Zhe Yu, Xin Guo, Quan Chen, Fei Zheng, Haigang Li, Wei Zhang, Tao Tang, Jiekun Luo, Yang Wang

**Affiliations:** 1 Institute of Integrative Medicine, Department of Integrated Traditional Chinese and Western Medicine, Xiangya Hospital, Central South University, Changsha, 410008 Hunan People’s Republic of China; 2 National Clinical Research Center for Geriatric Disorders, Xiangya Hospital, Central South University, Changsha, 410008 Hunan People’s Republic of China; 3 NATCM Key Laboratory of TCM Gan, Xiangya Hospital, Central South University, Changsha, 410008 Hunan People’s Republic of China; 4 Department of Neurology of Integrated Chinese Medicine, Xiangya Jiangxi Hospital, Central South University, Nanchang, 330006 People’s Republic of China; 5 The College of Integrated Traditional Chinese and Western Medicine, Hunan University of Chinese Medicine, Changsha, 410208 Hunan People’s Republic of China; 6 Hunan Key Laboratory of the Research and Development of Novel Pharmaceutical Preparations, Changsha Medical University, Changsha, 410219 Hunan People’s Republic of China

**Keywords:** Traditional Chinese medicine, Bioactive constituents, Network pharmacology, Metabolomics, Angiogenesis, PI3K/Akt/mTOR signaling pathway

## Abstract

**Background:**

Xuefu Zhuyu decoction (XFZYD) has been extensively utilized to treat traumatic brain injury (TBI). However, the bioactive compounds and the underlying mechanisms have not yet been elucidated.

**Objectives:**

This study aimed to investigate the bioactive constituents of XFYZD that are absorbed in the blood and the mechanisms in treating TBI.

**Methods:**

The study presents an integrated strategy in three steps to investigate the material basis and pharmacological mechanisms of XFZYD. The first step involves: (1) performing metabolomics analysis of XFZYD to obtain the main functions and targets; (2) screening the blood-entry ingredients and targets of XFZYD from databases; (3) obtaining the potential components targeting the key functions by integrated analysis of metabolomics and network pharmacology. The second step involves screening pharmacological effects with active ingredients in vitro. In the third step, the effects of the top active compound were validated in vivo, and the mechanisms were explored by protein antagonist experiments.

**Results:**

Metabolomics analysis revealed that XFZYD treated TBI mice mainly through affecting the functions of blood vessels. We screened 62 blood-entry ingredients of XFZYD by network pharmacology. Then, we focused on 39 blood-entry ingredients related to vascular genes enriched by XFZYD-responsive metabolites. Performing the natural products library, we verified that hydroxysafflor yellow A (HSYA), vanillin, ligustilide, paeoniflorin, and other substances promoted endothelial cell proliferation significantly compared to the control group. Among them, the efficacy of HSYA was superior. Further animal studies demonstrated that HSYA treatment alleviated neurological dysfunction in TBI mice by mNSS and foot fault test, and decreased neuronal damage by HE, nissl, and TUNEL staining. HSYA increased the density of cerebral microvessels, raised the expression of angiogenesis marker proteins VEGFA and CD34, and activated the PI3K/Akt/mTOR signaling pathway significantly. The angiogenic effects disappeared after the intervention of PI3K antagonist LY294002.

**Conclusion:**

By applying a novel strategy of integrating network pharmacology of constituents absorbed in blood with metabolomics, the research screened HSYA as one of the top bioactive constituents of XFZYD, which stimulates angiogenesis by activating the PI3K/Akt/mTOR signaling pathway after TBI.

## Introduction

Traumatic brain injury (TBI), resulting from external mechanical forces, has emerged as the leading cause of global mortality and disability [1, 2]. TBI causes severe deficits in motor, sensory, and cognition functions, exerting a substantial impact on the overall well-being of survivors. However, satisfactory treatment is not yet available [3].

Traditional Chinese medicine (TCM), rooted in a unique and comprehensive theoretical framework, is essential in the management of TBI. It is characterized by multi-component, multi-pathway, and multi-target therapy. As a typical traditional formula, Xuefu Zhuyu decoction (XFZYD) has been extensively employed in treating TBI to remove blood stasis in East Asia [4]. Modern studies have validated that XFZYD effectively alleviates symptoms and safeguards neurons from damage following TBI [5]. Previous studies showed that XFZYD improves neuro-functions of TBI through multi-mechanisms, including modulated miRNAs [6], tsRNAs [7], and affected axon guidance, amino acids metabolism [8] and angiogenesis [9]. However, due to the diversity of XFZYD ingredients and their complex interactions with the human body, research into the active components and pharmacological mechanisms of XFZYD in TBI healing remains limited. This has greatly hindered the broader application of XFZYD and the development of XFZYD-based modern medicines [10].

To explore the pharmacodynamic material basis, scientists have performed network pharmacology combined with computational analysis of physicochemical and absorption, distribution, metabolism, excretion, and toxicity (ADMET) properties [11, 12], and established the normative evaluation standard of network pharmacology [13]. Potential active ingredients were obtained through predicted oral bioavailability (OB) and drug similarity (DL) values, and then targets or biological processes were predicted for experimental verification. This strategy is established on the computational calculation, which may not truly reflect the in vivo activity of compounds. With the development of network pharmacology, blood-entry compounds were detected in vivo and were collected in some databases [14]. Based on the real detected blood-entry components, combined with screening and verification of the efficacy of active ingredients, more accurate and efficient results can be obtained.

**Fig. 1:**
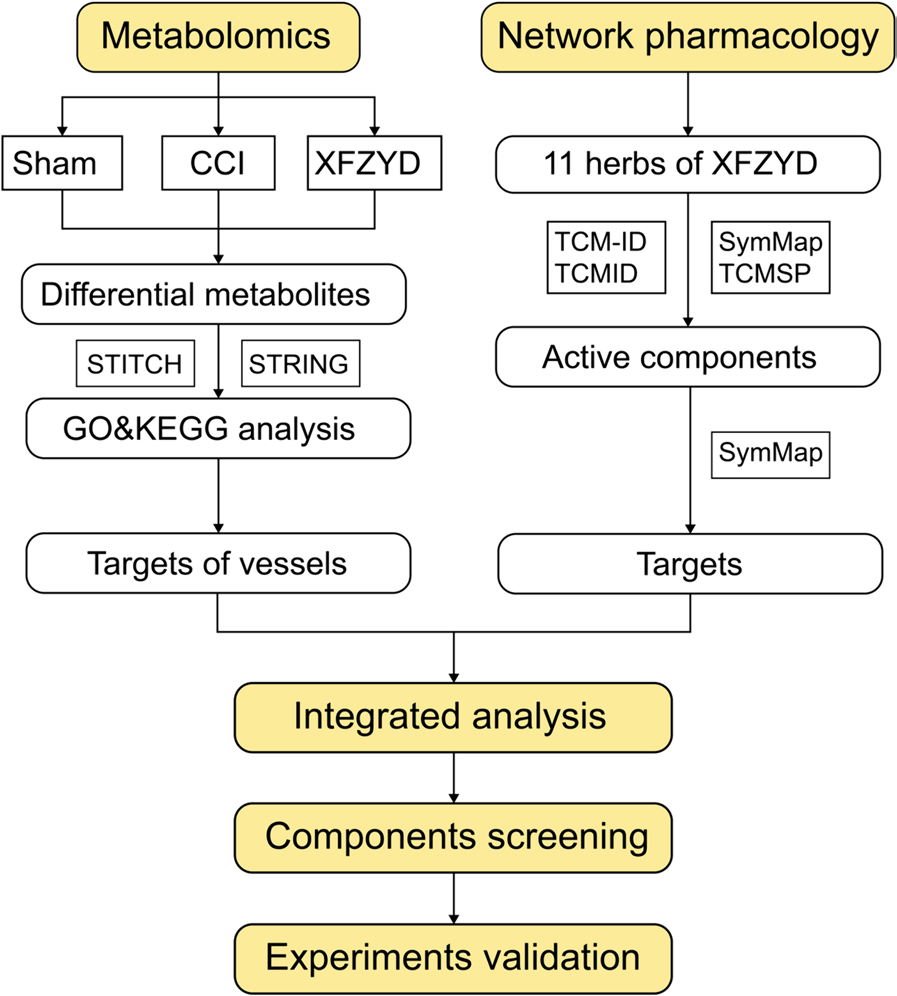
Subject design flowchart

This study presents an integrated strategy in three steps to investigate the material basis and pharmacological mechanisms of XFZYD. The first step involves: (1) performing metabolomics analysis of XFZYD to obtain the main functions and targets; (2) screening the blood-entry ingredients and targets of XFZYD from databases such as TCMSP, TCM-ID and SymMap; (3) the integrated analysis of (1) and (2). The second step involves screening for pharmacological effects with active ingredients in vitro using the natural products library. In the third step, the top active compounds were validated through further experiments (Fig. 1). This study provides a paradigm for the pharmacodynamic material basis study, which can be promoted and practiced in other herbal medicines and natural products.

## Materials and methods

### XFZYD preparation and quality control

XFZYD is comprised of 11 herbs including *Semen Persicae* (Tao Ren), *Flos Carthami* (Hong Hua), *Radix Angelicae Sinensis* (Dang Gui), *Radix Rehmanniae* (Sheng Di), *Radix Achyranthis Bidentatae* (Niu Xi), *Radix Paeoniae Rubra* (Chi Shao), *Fructus Aurantii* (Zhi Qiao), *Radix Glycyrrhizae* (Gan Cao), *Rhizoma Chuanxiong* (Chuan Xiong), *Radix Platycodonis* (Jie Geng), and *Radix Bupleuri* (Chai Hu), was sourced from the Pharmacy at Xiangya Hospital, Central South University, Changsha, Hunan. The formulation of XFZY involved mixing these herbs in a specific weight ratio of 8: 6: 6: 6: 6: 4: 4: 4: 3: 3: 2. This mixture was then processed into a freeze-dried powder, yielding an output of 16.9%. For application, the freeze-dried powder was reconstituted in purified water to achieve a final concentration of 0.095 g/mL.

Amygdalin, ferulic acid, and hydroxysafflor yellow A (HSYA) were purchased from Yuanye Bio-Technology Co., Ltd (Shanghai, China). Other reagents were analytically pure. The powder of XFZYD was vortexed with methanol and then separated on Waters UPLC HSS T3 column (1.8 μm, 2.1 × 100 mm) and followed by an AB API4000 LC/MS/MS (AB Sciex Pte. Ltd., USA) in negative ion mode.

### Animal experiments and ethics statement

The animal experiments received approval from the Medical Ethics Committee of Central South University and were fully compliant with the guidelines for animal care and use (Ethics approval number: CSU-2022-0321). All efforts were made to minimize animal suffering.

Sprague-Dawley male rats (8 weeks old, weighing 200–240 g) and C57BL/6 male mice (8 weeks old, weighing 20–28 g) were purchased from the Laboratory Animals of Central South University (Changsha, China). They were housed in specific pathogen-free (SPF) conditions under standard 12-h-light/dark cycles in rooms equipped with control for temperature (21 ± 1.5 °C) and humidity (50 ± 10%). The animals were given a one-week acclimatization period in the laboratory setting before initiating experiments.

### Establishment of the TBI model

The controlled cortical impact (CCI) model was established as previously published [15]. Rats were subjected to intraperitoneal administration of a 3% sodium pentobarbital solution, whereas mice were injected intraperitoneally with a 0.3% sodium pentobarbital solution. Both were then immobilized within a stereotaxic frame. The craniotomy was performed above the right hemisphere, midway between bregma and lambda. Brain injuries were induced in rats with a 5.0 mm depth, a velocity of 6 m/s, and a contact duration of 50 ms. For mice, the brain injuries involved a 1.5 mm depth, a velocity of 5 m/s, and a contact time of 100 ms. Concurrently, the sham group underwent identical craniotomy procedures but without any cortical injury. Following cranial closure, all animals were supine on a heating pad and monitored until they fully recovered from anesthesia.

### Experimental designs and drug administration

The animal study was separated into portions based on its goal: Part (1) To investigate the XFZYD-induced metabolic responses in the cerebral cortex of TBI on day 14, we randomly distributed rats into Sham, CCI, and XFZYD groups. Random numbers were produced using the standard = RAND () function in Microsoft Excel. The metabolomics were performed in Aksomics Biotechnology Co., Ltd. (Shanghai, China) with the number of animals not less than 10 per group. Hence, a minimal size of 10/group was used. The XFZYD group was administered 1.52 g/kg (equivalent to 9 g/kg of raw herbs), whereas the CCI and Sham groups were administered distilled water via gavage. Fourteen days post-treatment, the cortical tissue surrounding the injury site was excised and frozen in liquid nitrogen for subsequent metabolomic analysis. Part (2) To assess the effect of HSYA on angiogenesis following TBI. The mice were randomly assigned into four groups and maintained for 14 days: Sham group, CCI group, 10 mg/kg HSYA group, and 20 mg/kg HSYA. The sample size of animals was determined using a sample size calculator (http://www.lasec.cuhk.edu.hk/sample-size-calculation.html). On the condition that α = 0.05, 1−β = 0.9, the mNSS of TBI mice is 8.1 ± 0.8, while the treated group scores 6.6 ± 0.8. As a result, a minimal size of 6 mice per group was calculated. Part (3) To further elucidate the role of the PI3K/Akt/mTOR pathway in HSYA-induced angiogenesis in TBI, 30 min before craniotomy, mice were pre-treated with LY294002 (10 nmol/2 µL, Selleck, USA) as an antagonist of PI3K or DMSO through the Hamilton syringe at a rate of 0.667 µL/min [16]. On the condition that α = 0.05, 1−β = 0.9, the ratio of proteins for western blot in TBI mice is 1.5 ± 0.3, while the treated group scores 0.9 ± 0.2. As a result, a minimal size of 4 mice per group was calculated. To avoid experimenter bias, the operator conducting the neurological scoring was unaware of the grouping information.

### Animal dissection and tissue collection

On day 14 following modeling, all animals were internationally anesthetized with sodium phenobarbital (60 mg/kg). On the one hand, the rats and mice were perfused with 0.9% saline, and the cortex encompassing the region of brain injury was excised and preserved at − 80 °C until HPLC-MS/MS and western blot analysis. On the other hand, the rats and mice were subjected to left ventricular perfusion using a solution of 0.9% saline and 4% paraformaldehyde. Subsequently, the brains were taken out whole, soaked with para-formaldehyde, and sectioned with paraffin for staining.

### Metabolomics analysis

The cortex of rats was employed as the sample (*n* = 10 per group). Metabolite identification was conducted using the Agilent 1260 Infinity High-Performance Liquid Chromatography (HPLC) system (Agilent, CA, USA), linked with the Q-Exactive MS/MS (Thermofisher, MA, USA). The raw data files obtained from HPLC-MS/MS were processed using Compound Discover 2.1 (Thermofisher, MA, USA). Our methodology and parameters were consistent with those reported in our previous work [8]. For data analysis, we employed principal component analysis (PCA) and supervised orthogonal partial least squares discriminant analysis (OPLS-DA) using MetaboAnalyst version 5.0 (available at https://www.metaboanalyst.ca/). The integrity and reliability of our data were confirmed through cross-validation and permutation tests conducted 100 times. We identified differential metabolites using Student’s t-test, focusing on those with variable importance in projection (VIP) > 1 and *p* < 0.05. The results were visualized as heat maps generated using the MetaboAnalyst website (https://www.metaboanalyst.ca).

### Herbal-component-target network construction

We constructed the network by selecting metabolite-related genes from STITCH 5.0 (http://stitch.embl.de/). Using the UniProt database, these genes were then standardized to protein names (http://www.uniprot.org/). To identify vascular-related targets among these metabolite-related genes, we performed Gene Ontology (GO) and Kyoto Encyclopedia of Genes and Genomes (KEGG) pathway enrichment analysis using STRING 11.0 (https://string-db.org/). Following this, we focused on the blood components of XFZYD and their biological targets, which were extracted from the SymMap database (http://www.symmap.org). Utilizing this information, we constructed a visual network. This network depicted the blood ingredients of XFZYD and associated vascular targets, using Cytoscape for visualization and network analysis.

### Drug screening from natural products library

To verify the bioactive compounds in XFZYD targeting vessels, we acquired a natural products library from Selleck (Houston, USA) containing 2662 compounds. This library included 27 components identified in our screening: Vanillin, Paeoniflorin, Kaempferol, Nobiletin, 2-Methoxybenzoic acid, Apigenin, Citric acid, Liquiritin, Licochalcone A, Hydroxysafflor yellow A, Ligustilide, Hesperidin, Ferulic acid, Tetramethylpyrazine, Ononin, γ-Linolenic acid, Palmitic acid, Quinic acid, Licochalcone B, Stearic acid, Chlorogenic acid, Isoliquiritigenin, Oleic acid, Naringin, Glycyrrhizic acid, Amygdalin, Isovitexin. Each compound was dissolved in dimethyl sulfoxide (DMSO) and added to Brain-derived Endothelial cells.3 (Bend.3) lines at a concentration of 10 µM. After a 48-hour incubation period, cell viability was assessed using a CCK8 assay.

To evaluate the therapeutic effect of HSYA on CCI mice, we obtained HSYA with a purity of over 99% from Yuanye Pharmaceutical Co., Ltd. (Shanghai, China). HSYA was prepared at concentrations of 10 mg/kg and 20 mg/kg after dissolution and filtration.

### Cell culture and CCK8 assay

The Bend.3 cell lines were cultured in DMEM (Gibco, NY, USA), enriched with 10% fetal bovine serum (Oricell, Guangzhou, China) and 1% penicillin-streptomycin (Gibco, NY, USA). The cells were maintained in a humidified environment at 37 °C and 5% CO_2_. To evaluate cell growth, we employed the CCK8 assay. bEnd.3 cells were seeded in 96-well plates at a density of 1 × 10^3^ cells per well. Following overnight incubation, the cells were treated with the specified compounds. After 48 h of treatment, 10 µL of CCK8 solution was added to each well, and the plates were incubated for an additional hour at 37 °C. Absorbance was then measured at a wavelength of 450 nm to assess cell viability by a Microplate Reader.

### Behavioral testing

The modified neurological severity score (mNSS) was employed to assess the sensory, motor, and balance, and higher scores on the mNSS indicate worse neurological function. An elevated grid area measuring 80 cm square and composed of 25 mm square wire mesh was used for the foot fault test. Each mouse was placed individually on the wire mesh and allowed to move freely for 1 min while their movements were recorded on video. The results were calculated using the formula: [(contralateral faults − ipsilateral faults)/total steps] × 100%. Additionally, weight change was monitored, and weight was compared to its pre-modeling weight. These evaluations were conducted by an investigator who was blinded to the experimental groups. Assessments were carried out post-modeling on the 1st, 3rd, 7th, and 14th days.

### Hematoxylin–Eosin staining and nissl staining

The brain tissues were initially preserved using a 4% paraformaldehyde fix solution. Following fixation, the paraffin sections underwent a dewaxing process using xylene and were subsequently rehydrated by immersion in a series of graded ethanol solutions. Then, sections were stained with an HE staining kit (Servicebio, Wuhan, China) and Nissl staining solution (Servicebio, Wuhan, China). Digital images were obtained on the microscope (Axio Image M2, ZEISS, Germany) and measured by Image J.

### TdT-mediated dUTP nick end labeling (TUNEL) staining

TUNEL staining was conducted using a TUNEL apoptosis assay kit (Meilunbio, Dalian, China). The sections were costained with proteinase K for 5 min and incubated with TdT enzyme and FITC-12-dUTP for 1 h at 37 ℃. After rinsing, the sections were stained with DAPI (1:100, C0065, Solarbio) for 5 min and sealed with anti-fluorescence attenuation sealant. Finally, Images were obtained using a microscope (Axio Image M2, ZEISS, Germany) and digitized by ImageJ software.

### Fluorescein isothiocyanate (FITC)-dextran straining

As previously described, FITC-dextran staining was used to measure cerebral microvascular density [17]. Briefly, in a sheltered environment, the microvascular was shown by fluorography of the blood plasma after FITC-dextran (2 × 10^6^ molecular weight, Sigma-Aldrich, 50 mg/mL, 1 mL) was introduced into the vein in the tail and then sacrificed for 1 min. After the brain was removed, it was fixed in 4% paraformaldehyde and made into 30 μm frozen sections. Four fields of view around the cortical damage of each mouse were visualized by microscope (Axio Image M2, ZEISS, Germany) and calculated by Image J software.

### Immunofluorescence staining

As described previously, paraffin sections were antigen-repaired with citrate buffer. Next, the sections were blocked for 1 h with 3% BSA and 0.1% Triton X-100 in PBS, followed by incubation overnight at 4 °C with a rabbit anti-CD31 antibody (77699T, 1:1200, Cell Signaling Technology, Danvers, MA) and a mouse anti-PCNA antibody (2586, 1:3000, Cell Signaling Technology, Danvers, MA). After thorough PBS rinsing, the sections were exposed to Donkey anti-Rabbit Cy3-conjugated antibodies (1:1000, Jackson ImmunoResearch, West Grove, PA, USA) and Donkey anti-mouse AF488-conjugated antibodies (1:1000, Jackson ImmunoResearch, West Grove, PA, USA) for 1 h. Subsequently, the sections were stained with DAPI (C0065, 1:100, Solarbio, Beijing, China) for 5 min and sealed with anti-fluorescence attenuation sealant. Slices were visualized using a Zeiss Axio Imager M2 microscope.

### Western blot analysis

Proteins of cortex were extracted using RIPA lysis buffer, protease inhibitors, and phosphatase inhibitor (Beyotime, Shanghai, China) and quantified with a bicinchoninic acid (BCA) protein assay (Thermo Scientific, MA, USA). Equal amounts of protein were separated using 8–12% sodium dodecyl sulfate-polyacrylamide (SDS-PAGE) and transferred to polyvinylidene difluoride (PVDF) membranes (Millipore, Billerica, MA, USA). Next, the membranes were blocked with 5% skim milk for 2 h at room temperature and incubated overnight at 4 °C with different primary antibodies. On the second day, membranes were washed with Tris-buffered saline with Tween 20 (TBST) for 30 min, followed by incubation with the corresponding secondary antibody for 1 h. Furthermore, bands were detected with enhanced chemiluminescence (ECL) and ChemiDoc Touch (Bio-rad, CA, USA). In addition, to detect multiple antibodies on the same membrane, the exposed membranes were immersed in a Stripping buffer (Beyotime, Shanghai, China) for 30 min, and then the experiment, after the above transfer membranes step, was repeated. Lastly, the band intensities of the proteins were quantified with ImageJ software. Information on antibodies is as follows: CD34 (26233, 1:1000, CST), β-actin (3700 S, 1:1000, CST), VEGFA (YT5108, 1:1000, Immunoway), Phospho-Akt (4060T, 1:2000, CST), Akt (9272, 1:1000, CST), Phospho-mTOR (ab109268, 1:1000, Abcam), mTOR (ab32028, 1:1000, Abcam), Phospho-p70S6 kinase (YP0885, 1:1000, Immunoway), p70S6 kinase α (YT3555, 1:1000, Immunoway), HIF-1α (36169t, 1:1000, CST).

### Molecular docking

The 3D structure of HSYA was downloaded from PubChem Compound (https://www.ncbi.nlm.nih.gov/pccompound, PubChem CID: 6443665). The crystal structure of PI3K (PDB ID: 2Y3A) was acquired from the RCSB Protein Data Bank (https://www.rcsb.org/). HSYA and PI3K were converted to the pdbqt formats, which is compatible with AutoDock Vina [18]. Parameters for the docking pocket were set as follows: center coordinates (x = 32.014, y = − 46.29, _z = 2.791), dimensions (x = 50, y = 80, z = 120). Parameter of energy range = 5, number of docking modes generated (num_modes = 10). The result was visualized by PyMoL and Discovery Studio.

### Statistical analysis

All values are presented as mean ± standard deviation (mean ± SD). One-way ANOVA, followed by Dunnett’s multiple comparisons tests, was used to compare means among various groups. Statistical analysis was performed with GraphPad Prism 9.0 (San Diego, CA, USA). Statistically significant was defined as p-Values less than 0.05.

## Results

### XFZYD ameliorates neuronal damage in the lesion cortex following the CCI

**Fig. 2:**
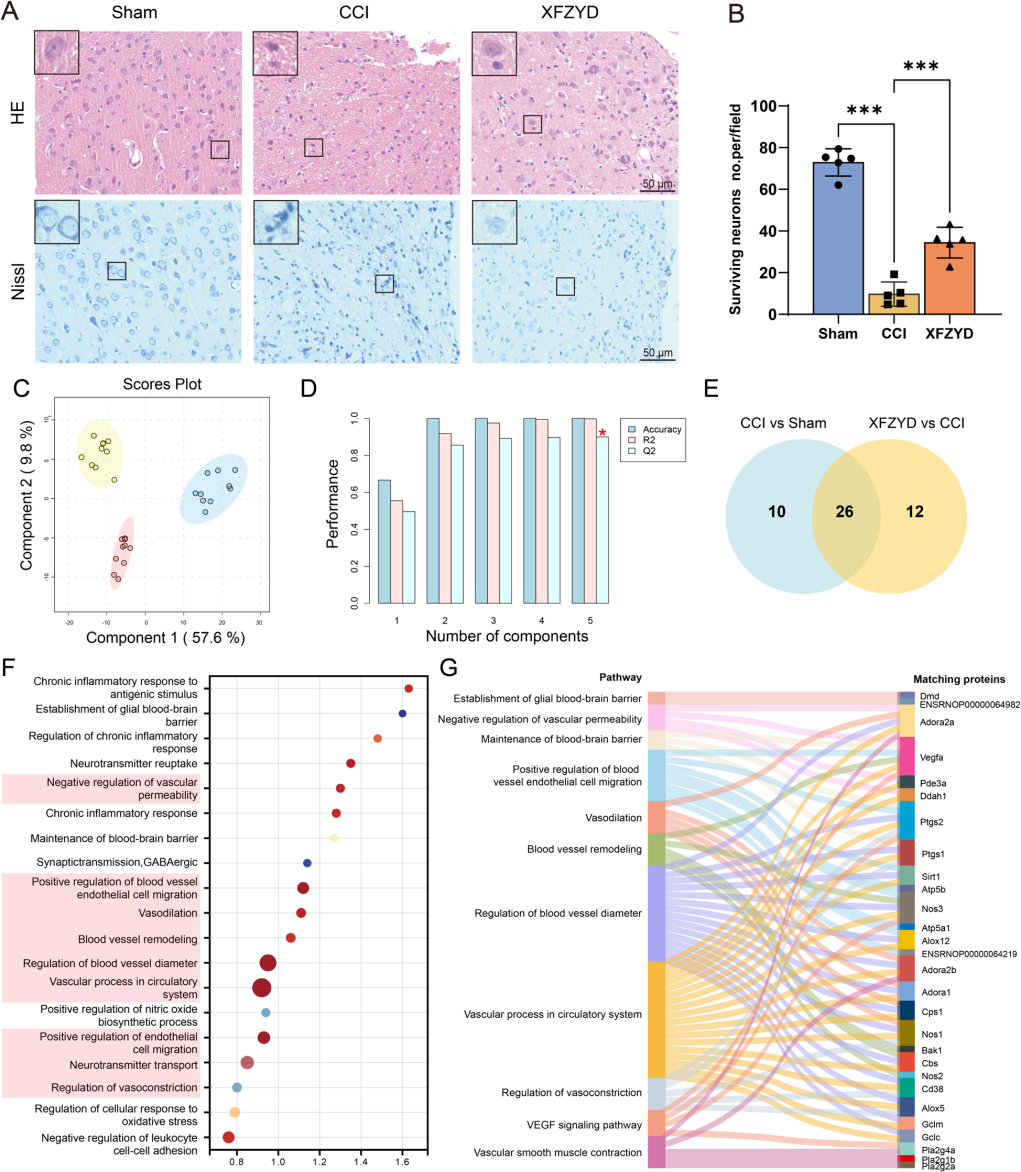
XFZYD modulates the metabolic profile of the cortex following TBI. **A** Illustration of cortical tissue using HE staining and Nissl staining. **B** Histograms illustrate the measurement of damaged neurons in the brain. *n* = 5. **C** The metabolomic profiles were clustered using PLS-DA. *n* = 10. **D** The validation plot of the PLS-DA model was generated from the cross-validation. **E** The Venn diagram shows that XFZYD governs 26 metabolites in the cortex. **F** GO biological processes functional enrichment analysis. **G** Chord diagram of the KEGG/GO pathways related to vessel and corresponding targets. ***p < 0.001

The quality control of XFZYD was shown in Fig. S1. HE staining (Fig. 2A) exhibited that cortical neurons in the control group exhibited typical morphology characterized by intact structure, uniformly distributed cytostome, and well-defined nucleoli. In contrast, cortical neurons in the CCI group displayed disordered arrangement, cellular swelling and rupture, and fuzzy boundaries at 14 days. Notably, XFZYD-treated groups demonstrated reduced levels of neuronal demise and tissue damage. Nissl staining (Fig. 2A and B) manifested that XFZYD significantly decreased the number of dark neurons compared to the CCI group.

### Metabolic analysis reveals the vessel-related effects of XFZYD in treating TBI

To offer a comprehensive examination of the metabolic profiles of the Sham, CCI, and XFZYD groups in the cortex, a partial least squares-discriminant analysis (PLS-DA) (Fig. 2C) was employed, revealing a dense clustering within each group and a distinct separation among the groups. The cross-validation analysis of PLS-DA (Fig. 2D) demonstrated favorable levels of predictability and credibility, as evidenced by R2 values = 0.98834 and Q2 values = 0.90068. As depicted in Fig. 2E, we found 26 differential metabolites using the criteria of VIP > 1 and *p* < 0.05. Hence, XFZYD modulated metabolic profiles in CCI rats.

To gain a deeper insight into the functions of differential metabolites, we identified genes associated with differential metabolites and performed GO analysis (Fig. 2F). XFZYD plays a vital role in regulating the biological processes related to blood vessels. Then, the biological processes and KEGG pathway interrelated with vascular were further displayed and were posited as potential effectors of XFZYD in the process of angiogenesis (Fig. 2G).

### Integrated analysis and components screening of XFZYD in TBI treatment

**Fig. 3:**
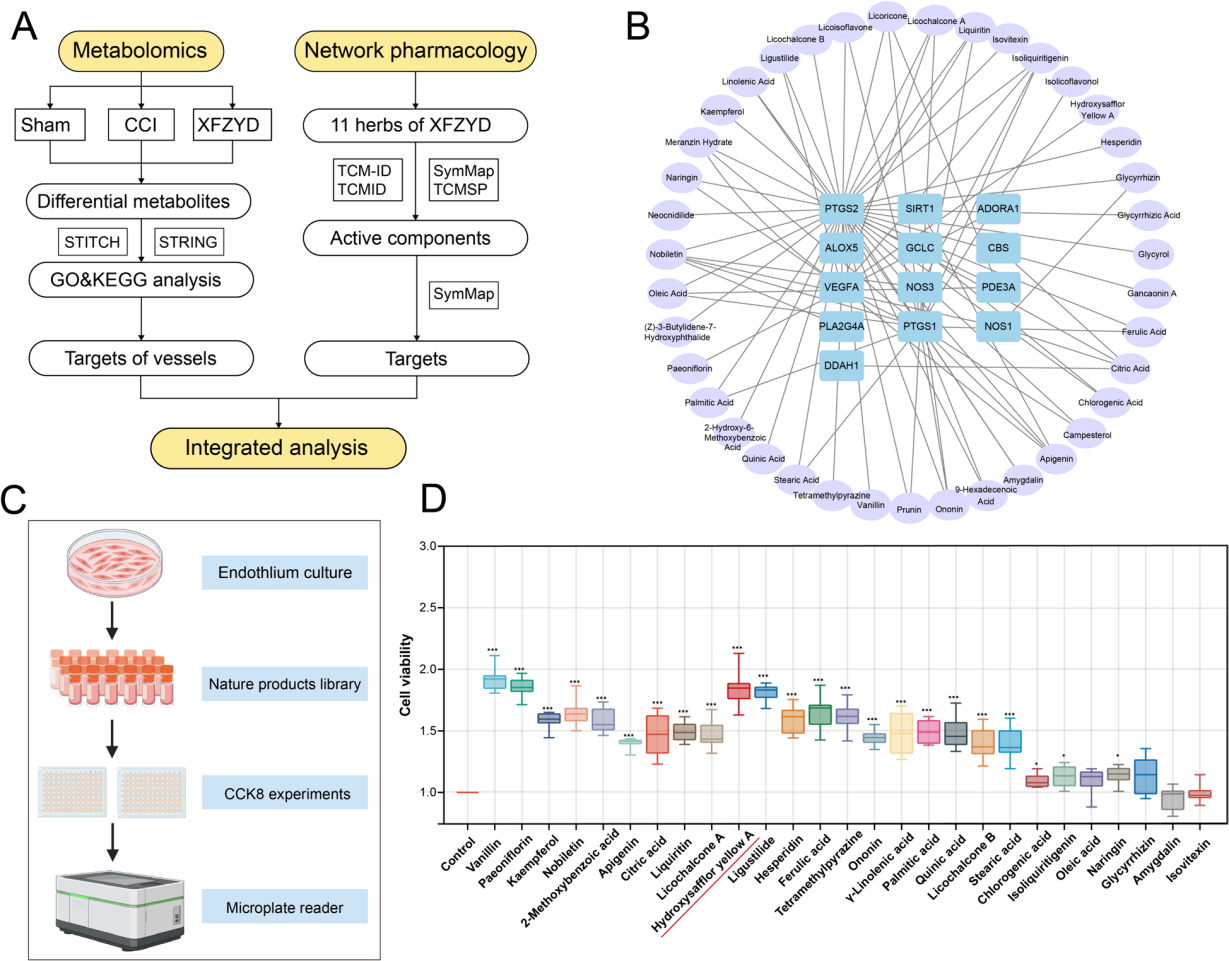
The integrated analysis and validation combined with metabolomics and network pharmacology of blood-entry constituents. **A** The integrated analysis. **B** Blood ingredient-target network. **C** The screening of active compounds using the natural products library. **D** The CCK8 experiments revealed pro-angiogenic compounds in XFZYD. *n* = 6, *p < 0.05, **p < 0.01, ***p < 0.001

In our quest to identify active components in XFZYD, we leveraged the SymMap database (integrates data from TCMID, TCM-ID, and TCMSP databases) for an exhaustive analysis of the constituents of the 11 Chinese herbs in XFZYD (Supplementary materials 1). Then, we conducted the integrated analysis as shown in Fig. 3A. We focused on 39 blood-entry ingredients related to vascular genes enriched by XFZYD-responsive metabolites (Fig. 3B). To verify the bioactive compounds in XFZYD targeting vessels, we acquired a natural products library from Selleck. New blood vessels are produced by the proliferation of endothelial cells. Hence, to assess the role of promoting endothelium proliferation, we employed the CCK8 assay on Bend.3 cells (Fig. 3C). The results revealed that HSYA, Vanillin, Ligustilide, Paeoniflorin, and other substances have a superior ability to promote endothelial cell proliferation compared to the control group (Fig. 3D). Among them, HSYA is the main component of XFZYD’s royal medicine safflower. Thus, we speculated that HSYA may be one of the main pro-angiogenic compounds in XFZYD.

### HSYA treatment ameliorated neurological deficits and neuron damage after TBI

**Fig. 4:**
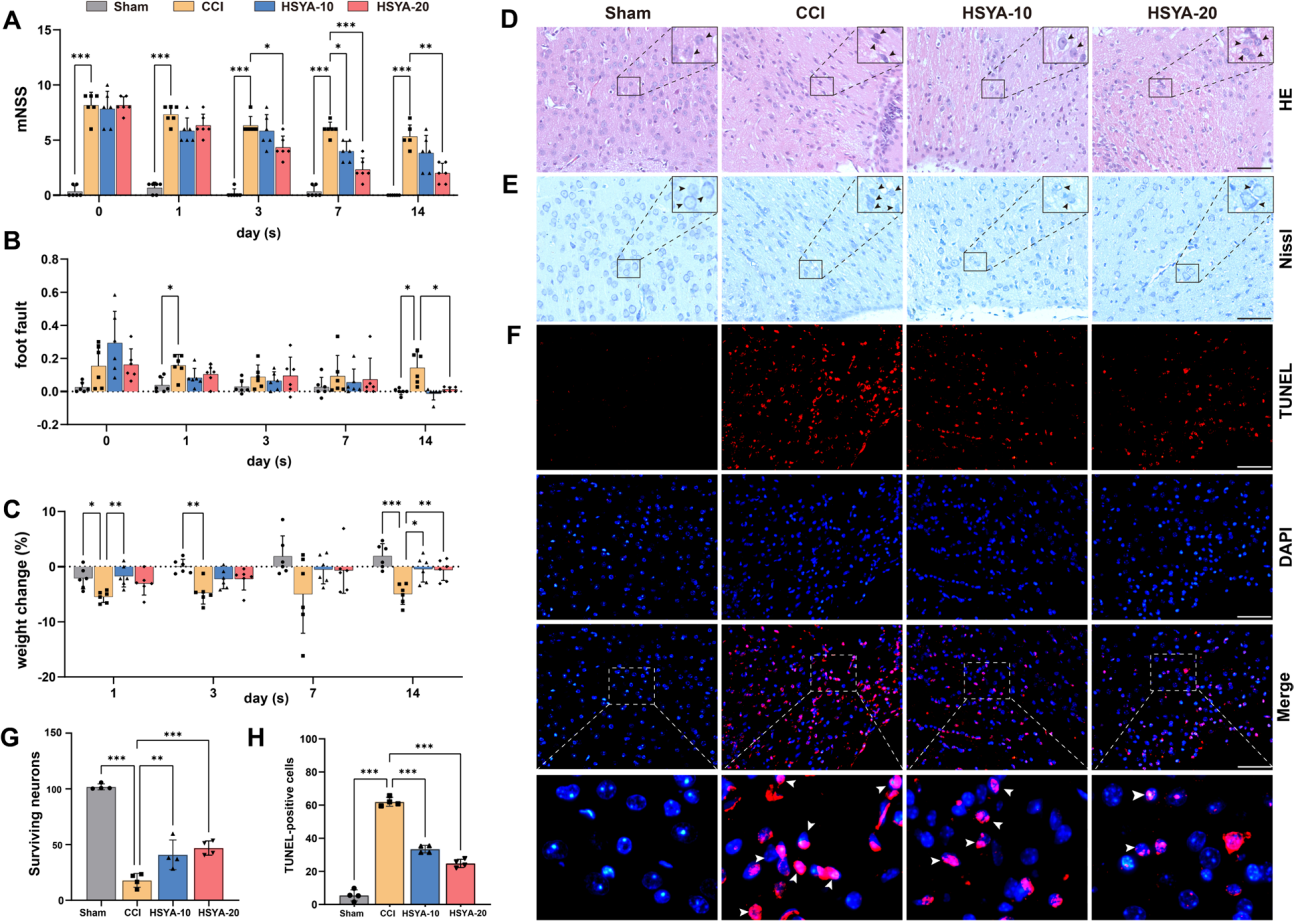
HSYA treatment ameliorated neurological deficits and neuron damage in TBI. **A** The neurological deficits were assessed by mNSS evaluation at 1, 3, 7, and 14 days after the operation, *n* = 6. **B** Sensorimotor function was measured with the foot fault at 1, 3, 7 and 14 days after the operation, *n* = 6. **C** Percentage change in body weight at 1, 3, 7, and 14 days, *n* = 6. Pathological changes in the cerebral cortex detected by HE (**D**) and Nissl staining (**E**) (×400, scale bars = 50 μm). **F** TUNEL staining (original magnification ×400; Red fluorescence: TUNEL; Blue fluorescence: DAPI; White arrows: TUNEL positive cells). **G** The quantification of surviving neurons was calculated by Nissl staining, *n* = 4. **H** Numerical representation of TUNEL-positive cells. scale bars = 50 μm, *n* = 4, *p < 0.05, **p < 0.01, ***p < 0.001

To investigate the therapeutic effect of HSYA, we conducted a series of behavioral tests on days 1, 3, 7, and 14, followed by histopathological assessments after day 14. As depicted in Fig. 4A and B, the mNSS test illustrated a significant recovery of neurological function with HSYA treatment, particularly 20 mg/kg HSYA on days 1, 3, 7, and 14. The foot fault test further demonstrated a reduction in the frequency of foot faults on day 14 with HSYA administration. HSYA administration resulted in faster weight repair than the CCI group during 14 days (Fig. 4C). H&E staining displayed that the cortical neurons in the CCI group were disordered, atrophied, and degenerated when compared to the sham group. Notably, such alterations could be reversed with HSYA treatment (Fig. 4D). Meanwhile, as revealed by nissl staining (Fig. 4E), HSYA therapy resulted in a notable reduction of dark neurons compared to the CCI group (Fig. 4G). TUNEL testing (Fig. 4F) confirmed HSYA restrained the CCI-induced increase in neuronal apoptosis on day 14 post-injury. Thus, the cumulative findings demonstrated that HSYA exerts a potent neuroprotective effect following TBI.

### HSYA stimulated angiogenesis in the cortex in TBI mice

**Fig. 5:**
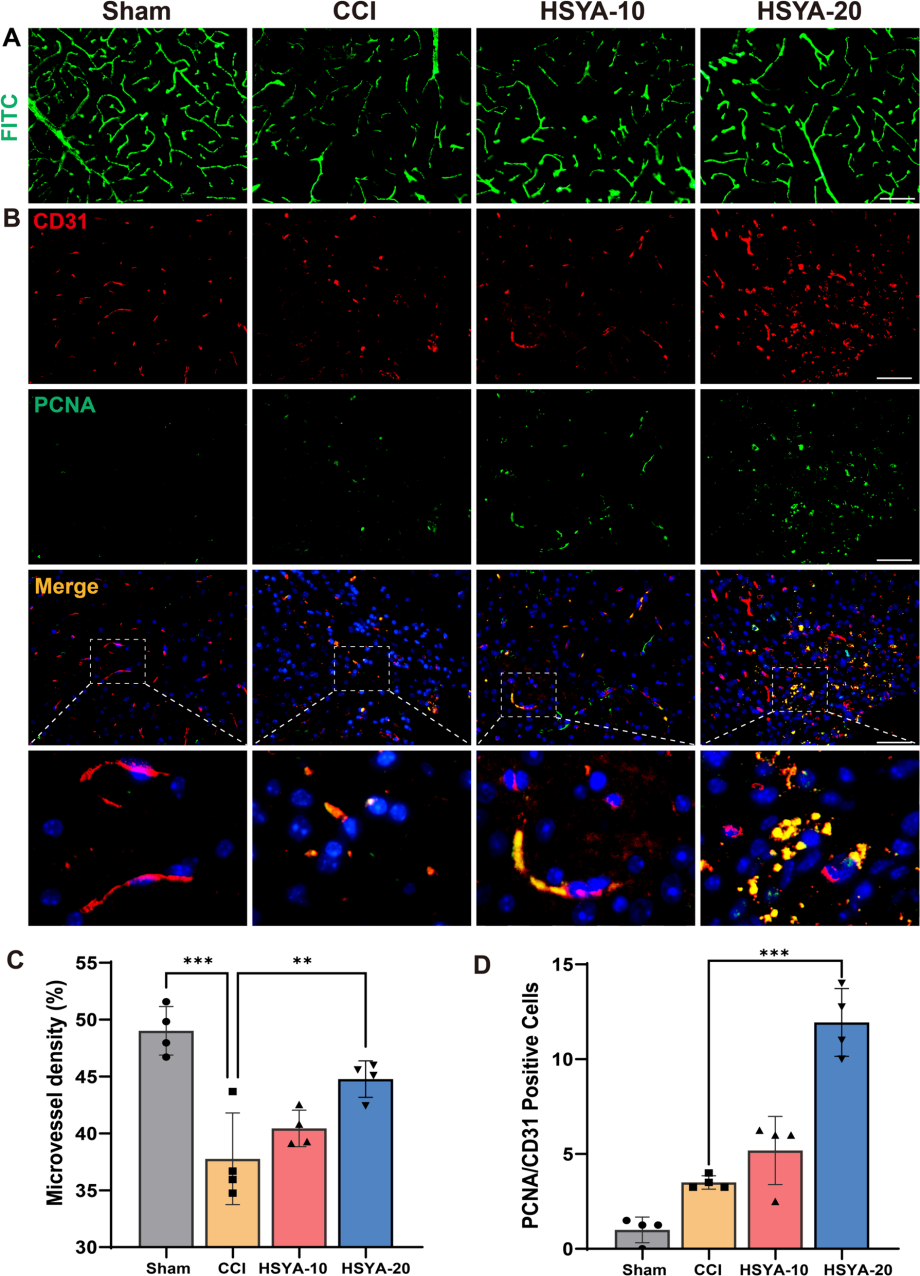
HSYA stimulated angiogenesis in the cortex following CCI. **A** Cerebral microvessels perfused with FITC-dextran and **C** quantification of vascular FITC-dextran fluorescence. **B** Representative immunofluorescence images of cerebral cortex sections stained for CD31 (red), PCNA (green), and DAPI (blue) with quantification of the PCNA/CD31 positive cells (**D**). scale bars = 50 μm, *n* = 4, *p < 0.05, **p < 0.01, ***p < 0.001

We investigated microvascular density utilizing FITC-dextran straining. The CCI group exhibited fewer microvessels integrated with FITC-dextran than the sham group. Notably, groups treated with HSYA, especially those receiving a concentration of 20 mg/kg, demonstrated a significant increase in FITC-dextran-integrated microvessels compared to the CCI group (Fig. 5A and C). Moreover, co-staining with the proliferation marker PCNA and the angiogenesis marker CD31 revealed that CCI led to a decrease in the formation of CD31-positive vessels, whereas HSYA treatment resulted in an increased formation of these vessels (Fig. 5B and D). It indicated that HSYA could stimulate the proliferation of endothelial cells.

### The angiogenic effect of HSYA was related to PI3K/Akt/mTOR signaling pathway

**Fig. 6:**
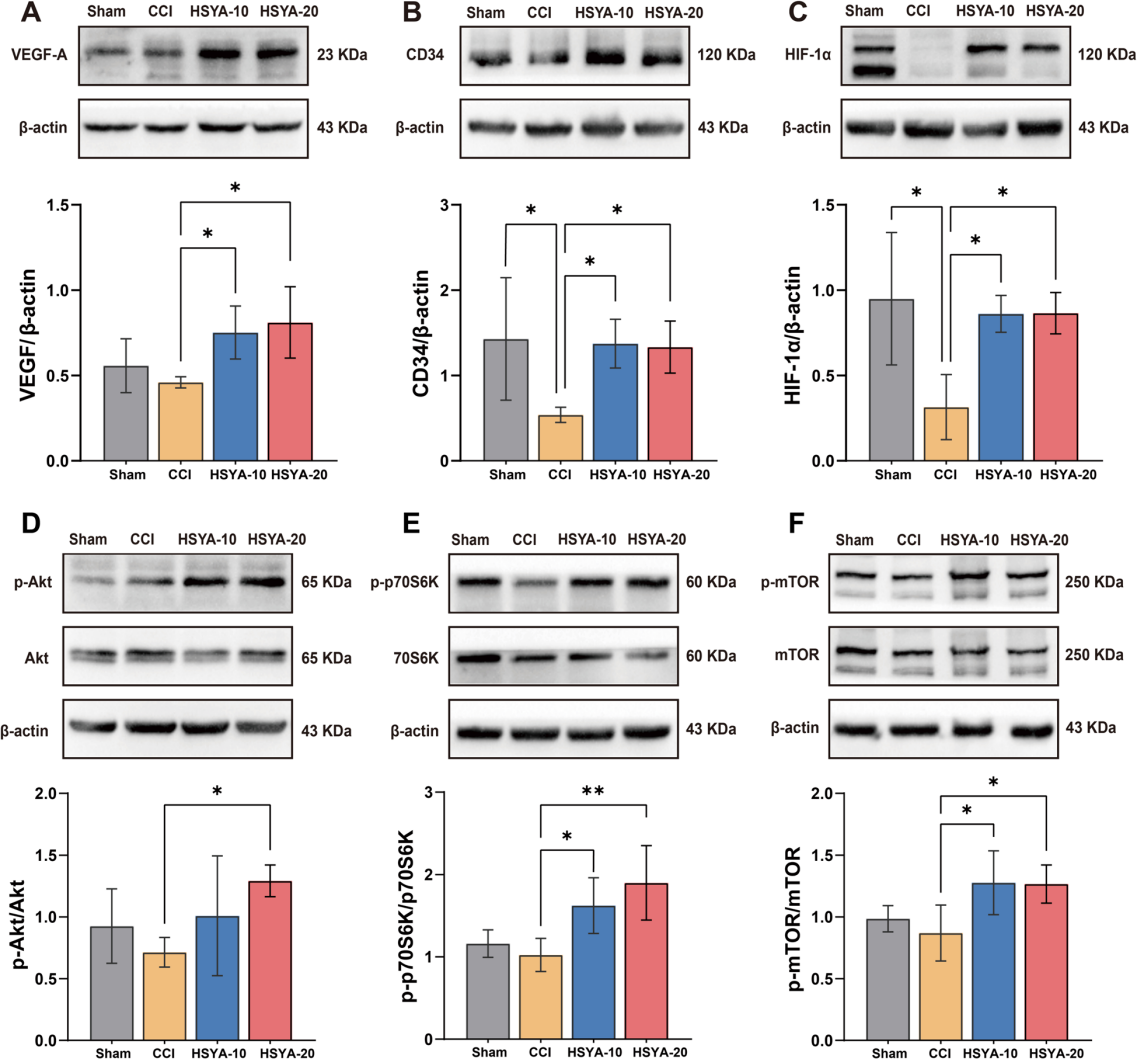
The angiogenic effect of HSYA was related to PI3K/Akt/mTOR signaling pathway. Representative western blots of VEGF-A (**A**), CD34 (**B**), and HIF-1α (**C**), phospho-Akt and total Akt (**D**), phospho-p70S6k and total p70S6k (**E**), phospho-mTOR and total mTOR along with their quantification in sham, CCI, 10 mg/kg HSYA, and 20 mg/kg on day 14. *n* = 4, *p < 0.05, **p < 0.01, ***p < 0.001

The impact of HSYA on angiogenesis was further explored using western blot analysis, focusing on key markers: VEGF-A, CD34, and HIF-1α. As is shown in Fig. 6A–C, VEGF-A, CD34, and HIF-1α were decreased in the cortex of the CCI group than in the Sham group. HSYA significantly increased their expression. Furthermore, 20 mg/kg HSYA demonstrated a more pronounced effect than the 10 mg/kg dosage. The regulation of VEGF and HIF-1α was closely related to the PI3K/Akt/mTOR signaling pathway [19]. Hence, we further explored the underlying mechanisms correlated to this pathway. Compared with the CCI group, HSYA activated p-Akt, p70S6k, and p-mTOR remarkably (Fig. 6D–F), especially the high concentration.

### HSYA promoted angiogenesis through activating the PI3K/Akt/mTOR signaling pathway after TBI

**Fig. 7:**
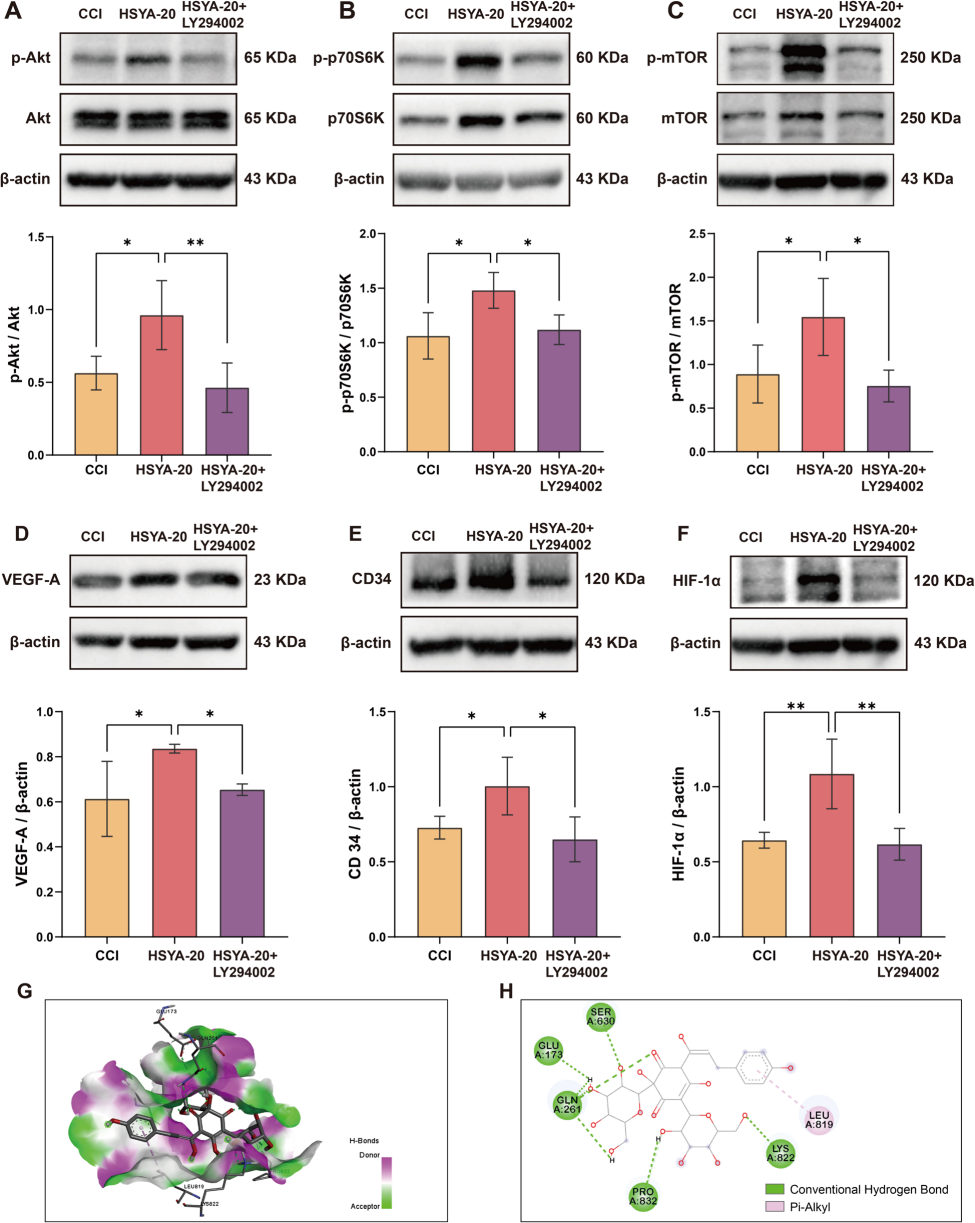
HSYA promoted angiogenesis through activating PI3K/Akt/mTOR signaling pathway in TBI. Western blotting showing the protein expression of **A** phospho-Akt and total Akt, **B** phosphor-p70S6k and total p70S6k, **C** phospho-mTOR and total mTOR, **D** VEGF-A, **E** CD34, and **F** HIF-1α in CCI, 20 HSYA, and 20 HSYA + LY294002. *n* = 4. Molecular docking of HSYA with PI3k in 3D (**G**) and 2D (**H**). *p < 0.05, **p < 0.01

To further investigate the role of the PI3K/Akt/mTOR pathway in HSYA-induced angiogenesis after CCI, we utilized the inhibitor LY294002 to suppress the PI3K. As shown in Fig. 7A–C, LY294002 significantly repressed HSYA-associated phosphorylation of Akt, p70S6k, and mTOR. Meanwhile, the expressions of the angiogenesis proteins VEGF-A, CD34, and HIF-1α were constrained by LY294002 (Fig. 7D–F). To further investigate the possibility of interaction between HSYA and PI3K, we applied molecular docking (Fig. 7G and H). A binding energy smaller than − 5 kcal/mol means a strong binding ability between ligands [20]. The binding energy of HSYA towards PI3K was − 9.1 kcal/mol, which indicated that HSYA may bind to PI3K and activate the PI3K/Akt/mTOR signaling pathway.

## Discussion

**Fig. 8:**
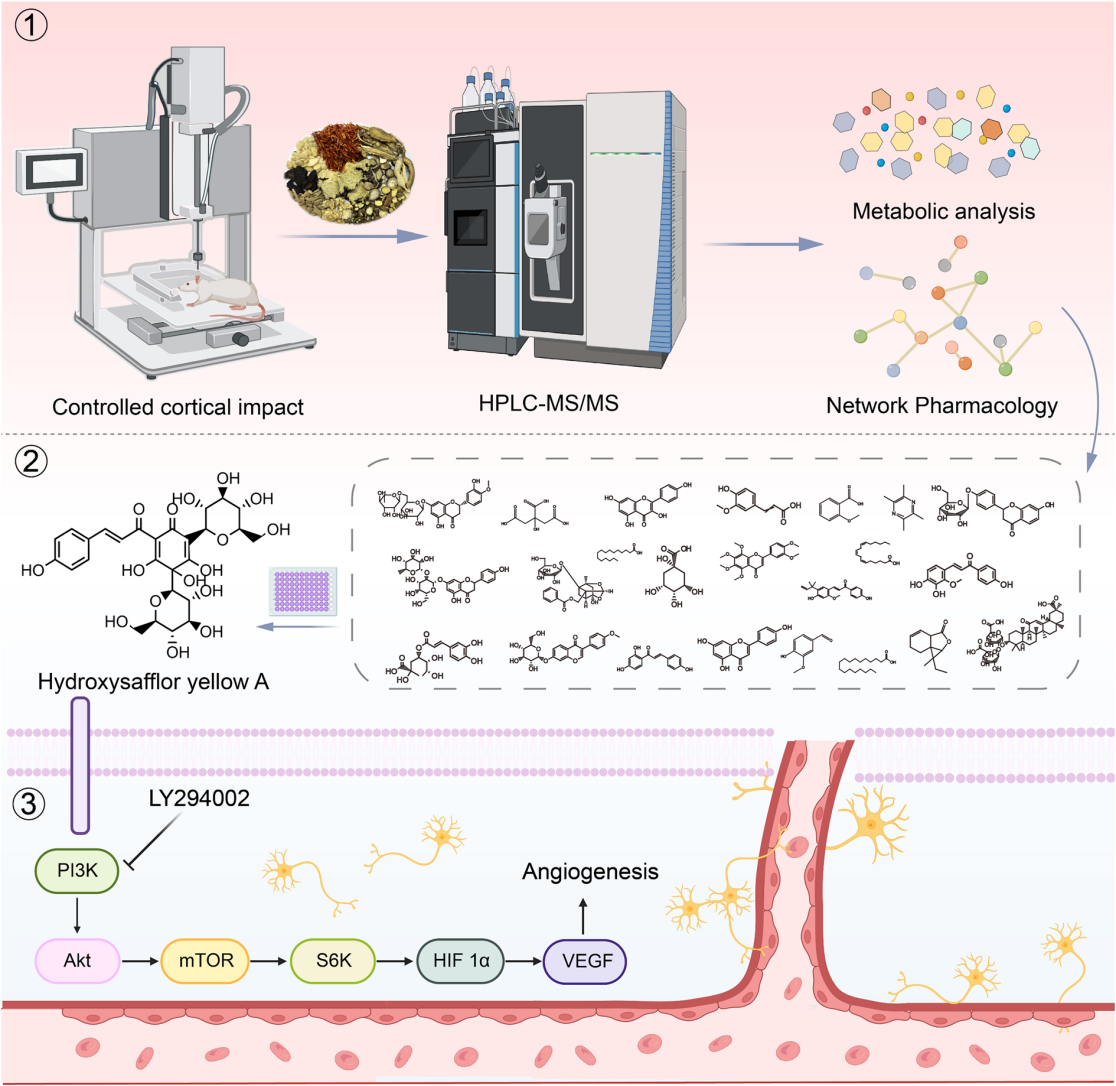
The integrated strategy of “three-steps”. Created with BioRender.com

The complexity of the formula makes the identification of the effective constituents and mechanisms challenging. Discovering the active constituents of TCM, assessing their efficacy, and elucidating the mechanism are key steps for interpreting the principles of TCM, and designing new modern drugs for treating diseases [21]. Guided by the distinctive theoretical system of TCM, XFZYD has been applied for thousands of years. Identifying its active ingredients and understanding its mechanisms are both urgent and essential. In our study, we developed a novel strategy that combined metabolomics and network pharmacology of blood-entry constituents to figure out the effective compounds of XFZYD and then screened the top compounds in vitro. It indicated that HSYA exerted the most prominent ability to boost endothelium growth. Furthermore, we demonstrated that HSYA promoted angiogenesis through activating the PI3K/Akt/mTOR signaling pathway. Molecular docking calculations verified the interaction between HSYA and PI3K. Our study explored the mechanisms of active ingredients in XFZYD, offering a valuable way to accelerate the development of new drugs (Fig. 8).

The primary injury generated by the mechanical force of TBI directly affects the meninges and parenchymal vessels. Clinical evidence shows that trauma-induced vascular injury after TBI is widespread and long-lasting in the injury area, accompanied by poor prognosis [22]. Early cerebrovascular injury events such as hemorrhage, edema, blood flow abnormalities, and blood-brain barrier disruption subsequently trigger other downstream processes such as inflammation, oxidative stress, altered metabolic substrate transport, and hypoxic and ischemic tissue damage. Although the body initiates angiogenesis in response to injury after TBI, the process is slow and the formed blood vessels have poor functionality, which is not enough to compensate for the vascular loss and dysfunction after TBI and cannot prevent the progression of TBI [23]. Promoting angiogenesis is essential for restoring cerebral damage produced by TBI, which enhances blood and oxygen delivery, diminishes immune cell infiltration, mitigates neuronal apoptosis, and facilitates neuronal remodeling [24, 25].

Of the 27 active ingredients we validated with Bend.3 cells, HSYA had the most robust angiogenic ability. HSYA is an active ingredient derived from the safflower. Some research studies have demonstrated that the brain tissue of CCI rats absorbed HSYA, exerted antioxidant effects by increasing SOD and CAT activities, GSH levels, and GSH/GSSG ratio, and promoted neurogenesis and axon regeneration through BDNF and STAT3/GAP43 axis [26–28]. However, there has been a lack of research investigating the potential impact of HSYA on the promotion of angiogenesis following CCI. In this study, we illustrated the efficacy of HSYA in enhancing neurological function and mitigating apoptosis utilizing the CCI mouse model. Furthermore, our findings revealed that HSYA administration promoted angiogenesis after TBI through western blot, FITC-dextran straining, and immunofluorescence staining. We also found that HSYA dose-dependently activated the PI3K/Akt/mTOR signaling pathway, which is significant in various cellular processes, encompassing angiogenesis, proliferation, survival, and metabolism [29, 30]. Consistent with this, intracerebroventricular injection of LY294002 reversed HSYA-induced Akt activation, which is phosphorylation of Akt, mTOR, and p70S6k, and inhibited HSYA -induced angiogenesis by reducing VEGF-A, CD34, and HIF-1α protein levels, indicating that HSYA promotes post-CCI angiogenesis through activation of the PI3K/AKT/mTOR pathway.

Other compounds also exert angiogenic effects, as reported previously. For instance, paeoniflorin is the main compound of Chi Shao. Paeoniflorin shows angiogenic actions on endothelial progenitor cells via up-regulating the expression of VEGF/VEGF-R2 in an ischemic stroke rat model [31]. Ligustilide is the main ingredient of Dang Gui and Chuan Xiong. It is a potential agent that improves angiogenesis and promotes recovery from ischemic stroke [32]. Kaempferol is a major flavonoid of Hong Hua. Kaempferol potentiates angiogenic functions in cultured endothelial cells by targeting VEGF [31]. Ferulic acid is the principal constituent of Dang Gui and Chuan Xiong, which promotes endothelial cell proliferation through up-regulating cyclin D1 and VEGF [33]. Hesperidin is one of the main components of Zhi Qiao. It enhances angiogenesis by modulating the expression of growth-related factors and inflammatory factors [34].

The blood-entry components of XFZYD also treat TBI through other mechanisms. Hence, we made a comprehensive analysis of existing research. Vanillin shows a neuroprotective effect by decreasing the level of lipid peroxidation and NO2, and inhibiting oxidative brain damage in TBI rats [35]. Bioinformatics reveals that ferulic acid is against TBI through anti-ferroptosis [36], targeting the MAPK signaling pathway and hypoxia-inducible factor-1 signaling pathway [37]. Apigenin exerts neuroprotective effects in TBI via regulating luminol and lucigenin and decreasing neuroinflammation [38, 39]. Kaempferol treatment significantly improves the TBI prognosis of rats during adolescence, with elevated neural connectivity, neurovascular coupling, and parenchymal microstructure in select brain regions [40]. Kaempferol also facilitates mitochondrial calcium uptake and improves cerebral blood flow and behavior after TBI [41]. Amygdalin attenuates neuroinflammatory injury via down-regulating toll-like receptors-2/toll-like receptors-4-nuclear factor kappa-B signaling pathway in vitro [42]. Tetramethylpyrazine treats TBI by reducing blood‑brain barrier permeability and enhancing peripheral cholinergic anti‑inflammatory effects [43], inhibiting neuronal apoptosis, alleviating oxidative stress damage [44], and attenuating periorbital allodynia [45]. Naringin alleviates cognitive impairment and improves the sensorimotor dysfunction of TBI rats by blocking neuroinflammation, oxidative stress, apoptosis, and regulating glutamate metabolism [46, 47]. Hesperidin attenuates depression-related symptoms in mice with mild TBI via decreasing neuroinflammation and oxidative damage, and enhancing BDNF production in the hippocampus [48]. Isoliquiritigenin protects against blood‑brain barrier damage and inhibits neuroinflammation [49, 50], attenuates oxidative stress-induced injuries via the Nrf2-ARE signaling pathway after TBI [51]. Glycyrrhizin blocks the detrimental effects of HMGB1 on inflammation and promotes cortical neurogenesis after TBI [52–54].

Our study provides an effective constituent discovery method of TCM. We used it to find the active substances of XFZYD and demonstrate the underlying mechanisms, which would assist the modernization of XFZYD and promote the discovery of innovative drugs for TBI treatment. Network pharmacology of blood-entry constituents selected the authentic effective TCM substances of blood from the administered animals and screened the effects by in vitro experiments, which avoided the influence of many factors and may reflect the effectiveness more accurately [55]. Top constituents were further verified by in vivo experiments to mimic the physiopathological and clinical features of patients, and to demonstrate the efficacy of constituents. This strategy combined herbs virtual screening, multi-target screening, and experimental screening and verification, which can be applied in the clinical practice of TBI to take the technological development from inefficient to efficient discovery, which is of great significance in innovative drug discovery of XFZYD. Furthermore, with the development of artificial intelligence technology [56], the application of massive omics data will help to reveal the complex treatment mechanism of TBI [57].

Our research has some limitations. Among the 39 components of XFZYD that were screened using metabolomics and network pharmacology, we solely focused on investigating the potential mechanism of action of HSYA. The remaining components warrant further exploration. In addition, some of the ingredients in XFZYD do not need to be absorbed into the blood to be effective. Some of them work by modulating intestinal flora. Therefore, gut microbiota metabolomics is needed. Moreover, other omics methods, such as genomics, transcriptomics, and proteomics, can be applied comprehensively to enrich this strategy and provide more accurate results.

## Conclusion

By performing a novel strategy of “three steps”, our study reveals that HSYA, a key active component of XFZYD, triggers angiogenesis of TBI mice through the PI3K/Akt/mTOR signaling pathway. Our research provides novel avenues for advancing research into the efficacious substance foundations of TCM, which may be applied in clinical practice to take the technological development from inefficient to efficient discovery.

## Data Availability

The data that support the findings of this study are available from the corresponding author upon reasonable request.

## Abbreviations

TBI: Traumatic brain injury
XFZYD: Xuefu Zhuyu decoction
HSYA: Hydroxysafflor yellow A
mNSS: Modified Neurologic Severity Score
TUNEL: TdT-mediated dUTP nick end labeling
FITC: Fluorescein isothiocyanate
ECs: Endothelial cells
SPF: Specific pathogen-free
GABA: Gamma-aminobutyric acid
DMSO: Dimethyl sulfoxide
Bend.3: Brain-derived endothelial cells.3
PCA: Principal component analysis
PLS-DA: Partial least squares-discriminant analysis
IHC: Immunohistochemistry
VEGF: Vascular endothelial growth factor
